# Chemical Characterization and Biological Activity of Astaxanthin Extracted from *Callinectes sapidus* By-Products: Implications for Oxidative Stress and Inflammatory Skin Disorders

**DOI:** 10.3390/ijms27093912

**Published:** 2026-04-28

**Authors:** Marco Casciaro, Roberta Tardugno, Filomena Corbo, Angelica Spano, Paola Lucia Minciullo, Eleonora Di Salvo, Sebastiano Gangemi, Nicola Cicero

**Affiliations:** 1 School and Operative Unit of Allergy and Clinical Immunology, Department of Clinical and Experimental Medicine, University of Messina, 98125 Messina, Italy; marco.casciaro@unime.it (M.C.); paola.minciullo@unime.it (P.L.M.); gangemis@unime.it (S.G.); 2 Department of Pharmacy—Drug Sciences, University of Bari Aldo Moro, 70125 Bari, Italy; roberta.tardugno@uniba.it (R.T.); filomena.corbo@uniba.it (F.C.); 3 Interdisciplinary Department of Medicine, University of Bari Aldo Moro, 70125 Bari, Italy; a.spano8@phd.uniba.it; 4 Department of Biomedical, Dental, Morphological, and Functional Image Sciences (BIOMORF), University of Messina, 98125 Messina, Italy; nicola.cicero@unime.it; 5 Meat and Agribusiness Chain Research Consortium, Polo Universitario dell’Annunziata, 98168 Messina, Italy; 6 Science4life Spin-Off Company, University of Messina, 98168 Messina, Italy

**Keywords:** *Callinectes sapidus*, blue crab, astaxanthin, by-product, marine waste, antioxidant activity, HPLC analysis, human health, skin diseases

## Abstract

Astaxanthin (AST) is a carotenoid with powerful antioxidant and anti-inflammatory properties and increasing interest in dermatological and nutraceutical applications. In this study, AST-rich extracts were obtained from by-products of the blue crab *Callinectes sapidus* and chemically characterized using HPLC-DAD analysis. The antioxidant activity of the extracts was assessed through complementary spectrophotometric assays (DPPH and FRAP). Comparable AST contents were detected in the two extracts, with values of 1.269 ± 0.006 and 1.219 ± 0.015 mg/100 g dry weight for EtOH and IPrOH, respectively. However, the EtOH extract exhibited higher antioxidant activity, reaching 0.10 ± 0.01 mg Trolox equivalents (TE)/g in the DPPH assay and 0.27 ± 0.02 mg TE/g in the FRAP assay, compared with 0.08 ± 0.01 and 0.11 ± 0.03 mg TE/g for the IPrOH extract. The biological activity of AST extracts was further evaluated against the opportunistic pathogen *Staphylococcus aureus* and beneficial lactic acid bacteria. AST exhibited moderate antibacterial activity against *S. aureus*, with an MIC value of 50 μg/mL and inhibition zones up to 14 mm at 200 μg/disc, while promoting the proliferation of *Lactobacillus plantarum*, *Lactobacillus casei*, and *Lactobacillus reuteri*. These findings highlight the prospective valorization of blue crab by-products as a sustainable supply of antioxidant and microbiota-modulating compounds with possible applications in skin health and cosmetic formulations.

## 1. Introduction

The blue crab, *Callinectes sapidus* (*C. sapidus*), is native to the western Atlantic coasts of the Americas. It is a highly adaptable euryhaline organism, capable of inhabiting both marine and brackish environments such as estuaries, lagoons, and coastal areas [1]. The ecological success of *C. sapidus* is related to a combination of biological and behavioral characteristics, including rapid growth, a highly flexible and opportunistic feeding strategy, elevated reproductive potential, and the capacity to exploit a wide range of habitats. Furthermore, its efficient larval dispersal and competitive survival strategies, such as scavenging and cannibalism, contribute to its invasive capacity [2]. To date, the blue crab is considered one of the most impactful invasive alien species in the Mediterranean Sea, where it exerts significant pressure on ecosystem biodiversity and negatively affects several human activities, including fisheries, aquaculture, and tourism [3]. Despite its negative ecological impacts, *C. sapidus* may represent a novel economic resource, given its high nutritional value and its potential as a viable alternative for the reuse of the carapace as a by-product. Indeed, the food industry produces a substantial quantity of by-products, such as carapace, meat residues, viscera, and legs, accounting for up to 85% of the total weight of blue crabs. This leads to the generation of thousands of tons of waste on a global scale [4]. This problem is particularly relevant in countries where a supply chain for recovery and valorization has yet to be established, with such waste often being disposed of through landfill or incineration. Consequently, the utilization and valorization of alternative crab by-products have been promoted through environmental awareness initiatives and regulatory frameworks [5,6].

**Figure 1:**
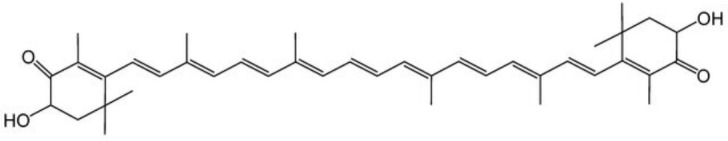
Astaxanthin chemical structure.

In recent years, increasing attention has focused on the recovery of high-value bioactive compounds from marine by-products within a circular economy framework. Among these, astaxanthin (AST), a xanthophyll carotenoid widely distributed in marine organisms, has attracted growing interest due to its pronounced redox-modulating activity [7,8]. In parallel, the growing demand for natural bioactive ingredients in the cosmetic and nutraceutical industries has further stimulated interest in AST-based formulations, particularly for skin health applications [9]. AST has been associated with a wide range of biological activities, including UV protection [10], skin depigmenting effects [11], and anticancer activity [12], as well as anti-inflammatory, immunomodulatory, and antioxidant effects [13]. Its unique polyene structure, characterized by conjugated double bonds and functional hydroxyl and keto groups, enables efficient scavenging of reactive oxygen species (ROS). It also modulates redox-sensitive signaling pathways (Figure 1) [14]. Oxidative stress is a key contributor to the pathogenesis of inflammatory skin disorders, such as atopic dermatitis (AD), where excessive ROS production leads to barrier impairment, dysregulated cytokine responses, and alterations in the skin microbiota [14,15,16]. In this context, AST has been reported to exert protective effects on the skin by promoting tissue repair and mitigating radiation-induced damage. These effects are mainly attributed to its ability to regulate oxidative stress, modulate inflammatory pathways, and support essential cellular processes, including keratinocyte proliferation and migration. Moreover, its antioxidant activity contributes to the protection of cellular components such as DNA, lipids, and proteins, thereby enhancing cell survival under stress conditions [17,18]. Several in vivo and clinical studies have also demonstrated that AST supplementation can improve skin elasticity, reduce signs of photoaging, attenuate inflammatory responses associated with skin disorders, support angiogenesis, and influence collagen deposition and remodeling, which are essential for proper skin repair [19,20].

Extracts derived from *Haematococcus pluvialis* (*H. pluvialis*), characterized by a high content of AST, represent a valuable source of bioactive compounds for health-related applications. In particular, AST-enriched extracts may offer a cost-effective alternative to purified AST while still providing significant protective effects for the skin against environmental and oxidative stressors [21]. Currently, most commercial AST is obtained from microalgae such as *H. pluvialis*; however, alternative natural sources and more sustainable extraction strategies, including green and innovative technologies, are increasingly being explored. Moreover, skin colonization by *Staphylococcus aureus* (*S. aureus*) is strongly linked to inflammatory exacerbations, while probiotic *Lactobacillus* species may contribute to microbiota homeostasis and barrier integrity [22]. Despite the growing interest in marine-derived AST, its levels in crustaceans are highly variable, influenced by factors such as species, anatomical part, dietary carotenoid intake, environmental conditions, and the specific extraction protocols employed [23]. In addition, limited studies have combined rigorous chemical validation of AST extracted from *C. sapidus* by-products with functional investigations addressing redox activity and microbiota-related mechanisms.

In this context, the integration of quantitative chemical analysis with microbiological evaluation is essential to assess both the composition and the functional biological activity of novel AST sources.

Therefore, the present study aimed to chemically characterize and quantify AST extracted from *C. sapidus* by-products through HPLC-DAD analysis and to investigate its antioxidant potential and biological activity in vitro. Specifically, the study evaluated the redox-modulating capacity of different solvent extracts using complementary antioxidant assays and explored their effects on selected probiotic strains (*Lactobacillus casei*, *L. reuteri*, and *L. plantarum*) and on the opportunistic pathogen *S. aureus*, which is frequently associated with cutaneous dysbiosis in several skin pathologies. By integrating chemical validation with functional assays, this study seeks to elucidate whether marine-derived AST-rich bioactive extracts may contribute to the modulation of oxidative stress and microbial imbalance, two key mechanisms underlying inflammatory skin disorders.

## 2. Results

### 2.1. Extraction of AST-Rich Extracts

AST-rich extracts were obtained from *C. sapidus* carapace using ethanol (EtOH) and isopropanol (IPrOH) as extraction solvents. The extraction efficiency was evaluated based on AST recovery. The EtOH extract yielded approximately 0.025 mg of AST from 2 g of starting material, while the IPrOH extract yielded approximately 0.028 mg from 2.3 g, indicating comparable extraction efficiency between the two solvents. As reported in the literature, the two solvents utilized were among the most effective for the extraction of AST. These solvents are suitable for use on crab exoskeletons, are straightforward to handle, facilitate the evaluation of the effect of solvent polarity on AST recovery, and extract bioactivity [24,25].

### 2.2. Identification and Quantification of Astaxanthin Content by HPLC-DAD

**Figure 2:**
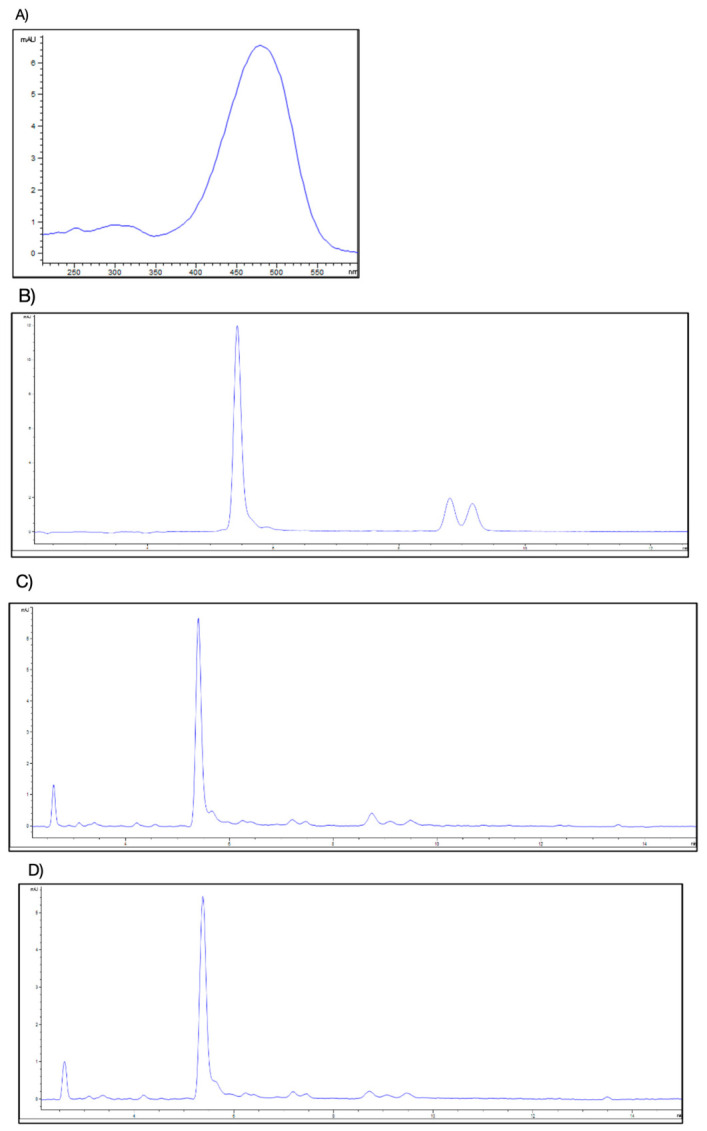
HPLC-DAD chromatograms: (**A**) UV-Vis spectrum of astaxanthin with maximum absorbance at 475 nm; (**B**) chromatogram of astaxanthin reference standard; (**C**) chromatogram of *C. sapidus* ethanol (EtOH) extract; (**D**) chromatogram of *C. sapidus* isopropanol (iPrOH) extract.

AST was identified in *C. sapidus* extracts by co-injecting the reference standard and eluting at 5.6 min in both EtOH and IPrOH extracts, showing the characteristic UV-Vis DAD absorption spectrum of astaxanthin with a maximum at 475 nm (Figure 2A). The presence of AST in both extracts was confirmed by HPLC-DAD analysis based on retention time and UV–Vis spectral characteristics (Figure 2B–D).

**Figure 3:**
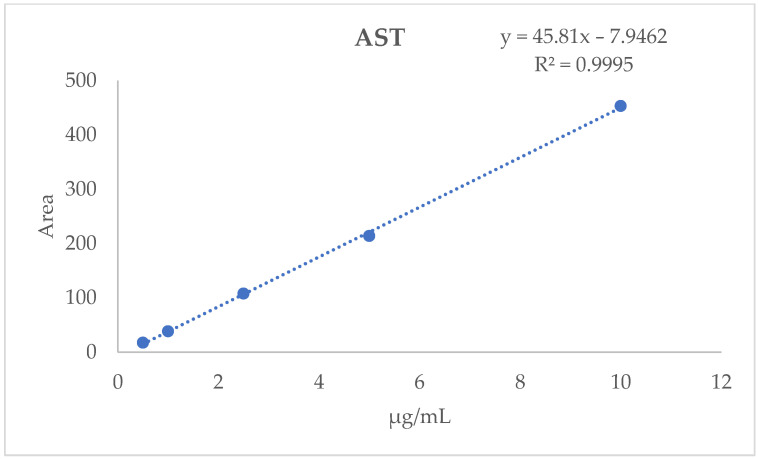
HPLC-DAD astaxanthin standard calibration curve.

Quantification was performed using an external calibration curve (y = 45.81x − 7.9462) with an optimal correlation coefficient (R^2^) of 0.9995 as reported in Figure 3.

The method showed high sensitivity, with a limit of detection (LOD) of 0.23 µg/mL and a limit of quantification (LOQ) of 0.70 µg/mL. The AST concentrations determined in the two different extracts are summarized in Table 1.

The results indicate that ethanol extraction provided a slightly higher yield (1.269 mg/100 g D.W.) compared to isopropanol (1.219 mg/100 g D.W.). This variation suggests a superior affinity of ethanol for the *C. sapidus* matrix or enhanced solubility of the carotenoid under the adopted experimental conditions.

### 2.3. Total Antioxidant Activity (TAA) of C. sapidus Extracts

The total antioxidant activity (TAA) of *C. sapidus* extracts was evaluated using DPPH and FRAP spectrophotometric assays [26,27,28]. As summarized in Table 2, the analyzed extracts exhibited antioxidant activity in both assays.

Regarding the DPPH results, the ethanol extract showed a higher antioxidant capacity (0.10 mg TE/g) compared to the isopropanol extract (0.08 mg TE/g). A similar trend was observed in the FRAP assay, where the ethanol extract demonstrated higher activity (0.27 mg TE/g) with respect to the isopropanol one (0.11 mg TE/g). In both DPPH and FRAP assays, ethanol exhibited a higher extraction efficiency of antioxidant compounds compared to isopropanol. This difference could be attributed to the higher polarity of ethanol, which may facilitate a better solubilization of polar and semi-polar antioxidant components. AST showed higher antioxidant activity than EtOH and IprOH extracts in both DPPH and FRAP assays, although the activity observed may suggest that the compound, as AST, when present in complex matrices, still contributes to the overall antioxidant potential. These results are consistent with those reported by Longo et al. [28], who demonstrated the presence of phenolic constituents and comparable antioxidant activity in an AST hydroalcoholic extract of the same species exoskeleton.

### 2.4. Effects of AST Extract on Bacterial Growth

For microbiological assays, AST was dissolved in DMSO and subsequently diluted in culture medium to obtain the desired concentrations in order to avoid interference from extraction solvents. The influence of AST extracts on bacterial growth was evaluated by monitoring the optical density (OD600) of cultures exposed to different extract dilutions (1:4, 1:8, 1:16, 1:32 and 1:64) over a 24 h incubation period. In contrast to the inhibitory effect observed for *S. aureus*, AST treatment promoted the growth of beneficial lactic acid bacteria, including *L. plantarum*, *L. casei*, and *L. reuteri*. For *L. plantarum*, AST exposure resulted in increased bacterial growth compared to the untreated control across all tested dilutions, reaching OD600 values significantly higher than the untreated control after 24 h. A similar trend was observed for *L. casei*, where AST exposure led to progressively higher OD600 values relative to the control. Likewise, *L. reuteri* exhibited enhanced growth in the presence of AST, particularly at higher dilutions, at 24 h compared with untreated cultures. Overall, these results indicate that AST extract exerts a selective bacteria-modulating effect, inhibiting the growth of the opportunistic pathogen *S. aureus* while promoting the proliferation of beneficial *Lactobacillus* spp.

### 2.5. Inhibition of S. aureus

**Figure 4:**
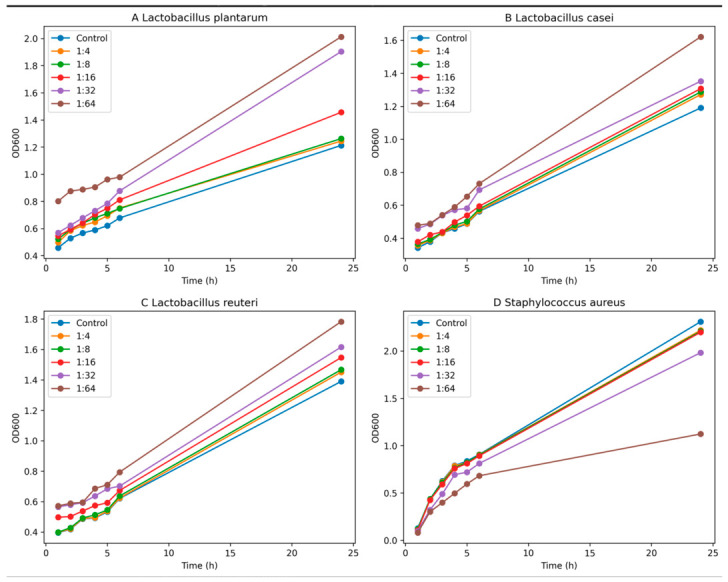
Growth kinetics of bacterial strains exposed to different dilutions of AST extract (1:4–1:64) measured as optical density at 600 nm (OD600) over 24 h. (**A**) *Lactobacillus plantarum*, (**B**) *Lactobacillus casei*, (**C**) *Lactobacillus reuteri*, and (**D**) *Staphylococcus aureus*. Untreated cultures were used as controls.

Growth kinetics analysis revealed that AST treatment affected the growth of *S. aureus* compared with the untreated control. During the early incubation phase (1–3 h), only minor differences between treated cultures and the control were observed (Figure 4). However, from 4 h onward, AST-treated cultures exhibited a progressive reduction in OD values. After 24 h, the inhibitory effect varied depending on the dilution applied, with the highest dilution (1:64) showing the most pronounced reduction in bacterial growth, corresponding to approximately 50% compared with the untreated control. Interestingly, although lower concentrations would generally be expected to result in reduced antibacterial activity, this behavior may be explained by improved dispersion of the extract and reduced interference from other matrix components at higher dilutions, which could enhance the effective availability of bioactive compounds. Intermediate dilutions (1:32) also produced noticeable inhibition, whereas lower dilutions (1:4–1:16) had only minor effects.

### 2.6. Antibacterial Activity of Astaxanthin Against S. aureus

The antibacterial activity of AST against *S. aureus* was assessed using the MIC assay. The MIC value for AST was determined to be 50 μg/mL, within the tested concentration range of 0.25–200 μg/mL. Although the inhibitory activity was lower than that of the reference antibiotic (amoxicillin), the extract showed measurable antibacterial effects at relatively low concentrations. The standard antibiotic amoxicillin, tested at a concentration of 0.25 µg/disk, showed an inhibition zone of 18 mm. The antibacterial activity was further evaluated using the disk diffusion assay (Table 3). AST produced measurable inhibition zones against *S. aureus* at concentrations equal to or higher than the MIC value. In particular, inhibition halos of 12 ± 0.04 mm, 12.7 ± 0.07 mm, and 14 ± 0.10 mm were observed at concentrations of 50, 100, and 200 μg/disk, respectively. No inhibition was detected at lower concentrations. These results confirm that AST extracted from *C. sapidus* waste exhibits moderate antibacterial activity against *S. aureus*. The AST-treated bacterial strains exhibited an inhibitory effect that varied depending on the dilution, as evidenced by a reduction in absorbance following the incubation period.

## 3. Discussion

The valorization of marine waste remains a pivotal issue, offering opportunities for economic valorization, particularly for applications related to human health. Evidence suggests that the exoskeletons of diverse marine invertebrates are plentiful in bioactive compounds, such as phenolic compounds, bioactive proteins and peptides, chitin, chitosan, carotenoids, lipids, and omega-3 fatty acids [29]. These compounds have gained considerable attention due to their broad spectrum of biological activities, including antioxidant, antimicrobial, anti-inflammatory, and immunomodulatory effects. Such properties make them promising candidates for applications in the nutraceutical, pharmaceutical, cosmetic, and agro-industrial sectors [30]. Indeed, the carapace of *C. sapidus*, which is generally considered to constitute a significant waste of marine resources, is a rich source of carotenoids, including AST [31]. The yield of AST in the blue crab carapace depends on several factors. The literature confirms that TAA should be assessed using at least two different assays to better characterize antioxidant reactivity. However, results can vary considerably across studies due to factors such as species-specific variations, habitat, and extraction parameters (e.g., solvent polarity, method, time, temperature, and solid-to-solvent ratio). Furthermore, the diverse units used to report TAA results often hinder direct comparison between studies, highlighting the need for standardized protocols and harmonized analytical methodologies to allow for a more reliable meta-analysis of bioactive compounds in crustacean by-products [23,27,28,32,33]. The antioxidant capacity observed in *C. sapidus* extracts was evaluated by two distinct chemical reaction mechanisms with the DPPH and FRAP assays. The relatively lower values observed for DPPH might be attributed to the complexity of the matrix and steric hindrance of the DPPH radical, which may limit the reaction rate with antioxidant chemical compounds [28,34]. The FRAP assay was employed to evaluate the reducing power of *C. sapidus* EtOH and iPrOH extracts. The reaction mechanism involved is a single-electron transfer (SET) involving the reduction of the ferric-tripyridyltriazine complex (Fe^3+^) to its ferrous form (Fe^2+^) at acidic pH. In both the spectrophotometric assays (DPPH and FRAP), the EtOH extract reported higher antioxidant activity compared to the isopropanol extract, whereas both extracts showed reduced activity compared to pure AST. Nevertheless, the lower antioxidant activity observed can be considered relevant, particularly given that they are derived from the carapace, a complex, unconventional waste-derived matrix with an antioxidant potential in agreement with the recent literature on this topic. Conversely, HPLC analyses showed comparable AST concentrations between the two extracts (1.27 vs. 1.22 mg/100 g for EtOH and iPrOH, respectively). These differences may indicate that AST is not the sole contributor to the antioxidant profile of the *C. sapidus* EtOH extract. The higher polarity of EtOH likely facilitated the co-extraction of additional electron-donor antioxidant constituents or prompted synergistic effects between these minor components and AST, thereby enhancing the reducing power. Further untargeted mass spectrometry screenings are required to identify these minor constituents [23,35]. A similar solvent-dependent behavior has been reported in previous studies on crustacean by-products. For instance, Karnila et al. [36] demonstrated that different acetone concentrations used for AST extraction from *Scylla serrata* shells resulted in significant variations in both extract yield and antioxidant activity. In particular, intermediate solvent polarity led to improved extraction efficiency and functional properties, highlighting the importance of solvent selection in modulating extract composition. These findings support the hypothesis that the antioxidant activity observed in the present study is not solely dependent on AST content but is also influenced by the chemical profile of the extract and the presence of additional bioactive compounds [36].

In addition, the skin hosts a complex microbial ecosystem that plays a key role in maintaining physiological homeostasis and protecting against external stressors. Alterations in this balance, commonly referred to as dysbiosis, have been associated with several dermatological disorders, often characterized by an overgrowth of opportunistic pathogens such as *S. aureus* and a reduction in beneficial bacterial populations, including *Lactobacillus* spp. [37,38,39]. In this context, the results obtained in the present study suggest that AST-rich extracts may exert a selective modulatory effect on bacterial populations. Specifically, AST showed inhibitory activity against the pathogenic strain *S. aureus* while simultaneously supporting the growth of probiotic lactic acid bacteria such as *L. casei*, *L. plantarum*, and *L. reuteri*, which are known for their beneficial role in maintaining skin microbial balance. This dual effect, characterized by inhibition of pathogenic bacteria and stimulation of beneficial strains, is particularly relevant and has been only partially explored in previous studies on marine-derived carotenoids [40]. Although the mechanisms underlying this selective effect remain to be clarified, it is possible that AST may influence bacterial growth through indirect mechanisms, such as modulation of oxidative stress or membrane interactions [41]. Similar effects have been described for other natural bioactive compounds, suggesting that complex interactions rather than direct antimicrobial activity alone may contribute to the observed behavior. These findings support the potential application of AST derived from *C. sapidus* waste as a functional ingredient for skin-related applications, although further studies are required to confirm its efficacy under physiological conditions.

These results indicate that AST-rich extracts derived from the carapace of *C. sapidus* could represent a valuable source of bioactive compounds with potential applications in cosmetic formulations and human health applications. Furthermore, the antioxidant properties of the extracts, together with their ability to inhibit *S. aureus* while supporting the growth of beneficial lactic acid bacteria, highlight their potential for use in the formulation of cosmeceuticals, nutraceuticals, and dietary supplements aimed at supporting skin health.

Nevertheless, the present study has some limitations. The biological activities were evaluated under in vitro conditions, and further in vivo studies are required to confirm the efficacy and safety of these extracts in physiological systems. Future studies integrating advanced extraction technologies, comprehensive chemical characterization, and biological evaluation will be crucial to better understand the mechanisms underlying the observed effects. Such multidisciplinary approaches may facilitate the valorization of marine by-products and support the development of innovative, sustainable health-promoting products.

## 4. Materials and Methods

### 4.1. Chemicals and Reagents

2,2-Diphenyl-1-picrylhydrazyl (DPPH), 2,4,6-tri(2-pyridyl)-S-triazine (TPTZ), Trolox (T), ethanol (EtOH), isopropanol (IPrOH), methanol (MeOH), acetone, sodium acetate (CH3COONa), ferric chloride (FeCl_3_), acid solutions, water (H_2_O), and acetonitrile (ACN) HPLC grade were obtained from Sigma-Aldrich (Milan, Italy). All-*trans* astaxanthin standard was purchased from Sigma-Aldrich (Milan, Italy).

### 4.2. Microorganism and Culture Preparation

In the present study, the bacterial strains *L. casei* and *L. plantarum* were isolated from a commercial probiotic yogurt drink, while the bacterial strains *L. reuteri* ATCC 55730 and *S. aureus* ATCC 25923, which have been certified, were purchased from Biogenerica Scientific Laboratory Supplies (Pedara, Italy). The *Lactobacillus* spp. strains were cultivated on MRS agar for 48 h at 37 °C, while the *S. aureus* strains were cultivated on Baird-Parker agar at 37 °C for 24 h. The test strains of bacteria were cultivated and maintained in a nutrient broth medium at 37 °C for 24 h.

### 4.3. Bacterial Growth

To ascertain the inoculum, the growth of the bacteria was monitored by measuring the optical density (O.D.) at 600 nm using a UV-VIS spectro-photometer (Fluo Star Omega, BMG Labtech, Ortenberg, Germany). The cells were diluted to achieve a viable count of 10^8^ CFU/mL. Accordingly, a 10% *v*/*v* cell suspension was utilized with the aim of constructing a calibration curve for each bacterial culture while simultaneously monitoring the correct growth of the bacterium throughout the experimental period.

### 4.4. Blue Crab Waste Preparation

In November 2023, specimens of *C. sapidus* originating from the Adriatic Sea (Italy) were purchased from a local fish market and transported to the laboratory at the University of Messina. The samples were obtained post-mortem, thereby avoiding any ethical issues associated with animal experimentation. Consequently, the crab sample was frozen at −20 °C. Prior to utilization, the crabs were defrosted in the fridge at 4 °C. Once the crab’s carapace has been separated from the rest of the body, it can be employed in applications requiring its use as a source of material. After that, the carapace was washed with distilled water and then dried in an oven for a period of 2–3 h at a temperature of 40 °C. Subsequently, the carapace samples were pulverized using a Mini-Prep Plus (Milan, Italy) and sifted through a standard Tyler series sieve set. The powder derived from the carapace of the crabs was prepared and stored at 4 °C in preparation for subsequent experiments. Dry weight values were calculated based on the initial dry mass of the starting material.

### 4.5. Astaxanthin Extraction

The extraction procedure was carried out following the method described by Dikel et al. [42] with minor modifications. Briefly, 2 g of carapace powder were extracted separately using 25 mL of isopropanol and 25 mL of ethanol in a 250 mL flask. The mixture was subjected to ultrasonic treatment using an ultrasonic bath (Merck, Darmstadt, Germany) operating at 40 kHz and 200 W for 1 h. Subsequently, the extracts were transferred into sealed tubes and incubated in a shaking water bath at 150 rpm for 24 h at room temperature. After extraction, solid residues were removed by centrifugation at 8000× *g* for 30 min. The supernatants were then collected and filtered through 0.45 µm membrane filters prior to further analysis.

### 4.6. HPLC-DAD Analyses

HPLC-DAD analyses were performed using an Agilent 1290 Infinity system (Agilent Technologies, Waldbronn, Germany) equipped with a vacuum degasser, a quaternary pump, a thermostated autosampler (maintained at 10 °C), a thermostated column compartment (25 °C), and a Diode Array Detector (DAD). Separation was achieved on an InfinityLab Poroshell 120 EC-C18 column (3.0 × 100 mm, 2.7 μm; Agilent Technologies) with an injection volume of 3 μL. The mobile phase consisted of water (A) and acetonitrile (B). The elution was performed at a flow rate of 0.5 mL/min using the following gradient program: 0–7 min, 90% B (isocratic); 7.1–12 min, linear gradient from 90% to 100% B; 12–15 min, 100% B (isocratic). A 10 min post-run time was applied for column re-equilibration. The DAD was operated in the 220–700 nm range, and the signal was monitored at 475 nm for quantification. Data were processed using Agilent OpenLAB software (version 1.3.1). Identification of AST was performed by co-injection with a reference standard. Quantitative analysis was carried out via external calibration using working standard solutions (0.5–10.0 μg/mL) prepared by serial dilution in acetone [43]. The resulting calibration curve (y = 45.81x − 7.9462, R^2^ = 0.9995) showed a limit of detection (LOD) of 0.23 μg/mL and a limit of quantification (LOQ) of 0.70 μg/mL. All analyses were performed in triplicate (*n* = 3) [27,44].

### 4.7. Total Antioxidant Activity (TAA)

#### 4.7.1. Determination of Radical Scavenging Activity (DPPH Assay)

The free radical scavenging activity of the extracts was evaluated spectrophotometrically using a BIOBASE Elisa microplate reader and 96-well microtiter plates (Greiner 96 Flat Bottom Transparent Polystyrene), following the method described by Gloria Bobo-García et al. [45]. Briefly, 20 μL of each sample was mixed with 180 μL of DPPH solution (150 μmol/L) prepared in methanol–water (80:20, *v/v*). The reaction mixtures were incubated in the dark at room temperature for 40 min, after which the absorbance was measured at 515 nm. Trolox was used as a reference standard (0.05–50 µg/mL) to construct the calibration curve (y = −6.526x + 0.5514, R^2^ = 0.9903). Results were expressed as milligrams of Trolox equivalents (TE) per gram of carapace powder sample (mg TE/g) by interpolating the absorbance values of the extracts with the Trolox standard calibration curve. All measurements were carried out in triplicate (*n* = 3), and data are reported as mean ± standard deviation.

#### 4.7.2. Determination of Ferric Reducing Antioxidant Power (FRAP Assay)

The antioxidant capacity of the extracts was determined spectrophotometrically using the FRAP assay, following the method described by Limongelli et al. [46], with slight modifications. Measurements were performed using a microplate reader (BIOBASE Elisa microplate reader) and 96-well microtiter plates (Greiner 96 Flat Bottom Transparent Polystyrene). Three reagent solutions were prepared: (a) acetate buffer (300 mM), obtained by dissolving 455.3 mg of CH_3_CO_2_Na·3H_2_O and 3.97 mL of CH_3_COOH in distilled water and adjusting the final volume to 250 mL; (b) TPTZ solution (10 mM), prepared by dissolving 156.2 mg of 2,4,6-tripyridyl-s-triazine in 0.17 mL of HCl (37% *v*/*v*) and diluting to 100 mL with distilled water; and (c) FeCl_3_·6H_2_O solution (20 mM), obtained by dissolving 270 mg of FeCl_3_·6H_2_O in 50 mL of distilled water. The FRAP working solution was prepared by mixing 25 mL of acetate buffer, 2.5 mL of TPTZ solution, and 2.5 mL of FeCl_3_·6H_2_O solution, followed by incubation at 37 °C for 30 min in the dark. Trolox standard solutions in methanol (0.003–0.15 mg/mL) were used to construct the calibration curve (y = 1.4147x + 0.0494, R^2^ = 0.9867). For the assay, 6 µL of each Trolox standard or sample extract was added to 194 µL of the working solution. After mixing, the reaction mixture was incubated in the dark at 37 °C for 15 min, and absorbance was measured at 593 nm. Results were expressed as milligrams of Trolox equivalents (TE) per gram of sample (mg TE/g). All measurements were performed in triplicate (*n* = 3), and data are reported as mean ± standard deviation.

### 4.8. Antimicrobial and Growth Modulation Assay

The antimicrobial activity of AST-rich extracts was tested against *S. aureus*, while their influence on the growth of probiotic bacteria (*L. casei*, *L. reuteri*, and *L. plantarum*) was also investigated. *S. aureus* was cultivated in Brain Heart Infusion (BHI) broth, whereas *Lactobacillus* spp. were grown in De Man, Rogosa, and Sharpe (MRS) medium. Bacterial suspensions were prepared in physiological saline, and their turbidity was adjusted to 0.5 McFarland standard by measuring the optical density at 600 nm (OD600) with a spectrophotometer. Prior to biological testing, extraction solvents were removed under reduced pressure using a rotary evaporator (Merck, Germany), and the dried extracts were subsequently dissolved in dimethyl sulfoxide (DMSO) to prepare stock solutions [47]. This step was performed to eliminate any potential interference of extraction solvents (ethanol or isopropanol) on bacterial growth, ensuring that the observed biological effects could be attributed exclusively to the extracted compounds. In addition, the use of DMSO allowed the preparation of homogeneous and reproducible stock solutions suitable for microbiological assays. For each assay, 10 µL of extract solution was added to 190 µL of culture medium containing the standardized bacterial inoculum, reaching a final volume of 200 µL per well in sterile 96-well microtiter plates. Under these experimental conditions, the final DMSO concentration did not exceed 2% (*v*/*v*). Control wells included bacterial cultures without extract (growth control) and sterile medium without inoculum (negative control), while an antibiotic was used as a positive inhibition control.

Plates were incubated at 37 °C for 24 h, and bacterial growth was monitored by measuring OD600 using a microplate reader (Fluo Star Omega, BMG Labtech, Ortenberg, Germany). Optical density readings were collected at hourly intervals during the first 6 h of incubation, followed by a final measurement at 24 h to assess both early growth kinetics and overall bacterial proliferation. OD values were converted into log_10_ cells/mL using previously established calibration curves correlating OD600 with bacterial concentration under identical experimental conditions. All experiments were performed in triplicate, and the results were expressed as mean values.

#### 4.8.1. Minimum Inhibitory Concentration (MIC)

The MIC of AST-rich extracts against *S. aureus* was determined using the microdilution method as previously described by Aribisala et al. [48], with minor modifications. Serial two-fold dilutions of the extract and the reference antibiotic (amoxicillin) were prepared in broth medium to obtain concentrations ranging from 200 µg/mL to 0.5 µg/mL. Subsequently, 100 µL of each dilution was distributed into the wells of a sterile 96-well microtiter plate, followed by the addition of 100 µL of standardized bacterial inoculum (approximately 10^6^ CFU/mL). Plates were incubated at 37 °C for 24 h, and bacterial growth was evaluated by visual inspection of turbidity. The MIC was defined as the lowest concentration of extract or antibiotic that completely inhibited visible bacterial growth.

#### 4.8.2. Disk Diffusion Method

The antibacterial activity of the extracts against *S. aureus* was further assessed using the disk diffusion assay. Briefly, bacterial cultures were grown in Mueller–Hinton (MH) broth and standardized to approximately 10^6^ CFU/mL. The suspension was uniformly spread onto Mueller–Hinton agar plates using a sterile cotton swab. Sterile paper disks (6 mm in diameter) were impregnated with different concentrations of AST extract and placed on the surface of the inoculated plates. The plates were incubated at 37 °C for 24 h. Following incubation, the diameters of the inhibition zones around each disk were measured in millimeters. The appearance of a clear inhibition zone was considered indicative of antibacterial activity.

### 4.9. Statistical Analysis

All experiments were carried out in triplicate, and data are presented as mean values ± standard deviation (SD). Statistical evaluation was performed using one-way analysis of variance (ANOVA), followed by Tukey’s post hoc test to assess differences among groups. A *p*-value lower than 0.05 was considered statistically significant.

## 5. Conclusions and Perspective

In conclusion, the present study demonstrates that the carapace of the blue crab *C. sapidus* may represent a valuable source of bioactive compounds, particularly AST, which can be recovered from this invasive species through sustainable valorization strategies. The obtained extracts exhibited measurable antioxidant activity and showed a selective biological effect on bacterial populations, inhibiting the growth of *S. aureus* while promoting the proliferation of beneficial lactic acid bacteria. Although ethanol and isopropanol extracts showed comparable AST contents, their antioxidant activities differed, with the ethanol extract displaying the highest activity in both DPPH and FRAP assays. In contrast, pure AST exhibited markedly stronger antioxidant activity than both extracts, suggesting that the overall bioactivity of the extracts depends not only on AST content but also on the contribution of co-extracted compounds and possible synergistic interactions. These findings highlight the potential of blue crab by-products as a source of natural compounds with possible applications in nutraceutical and cosmetic formulations aimed at supporting skin health. Moreover, this study contributes to the growing interest in the reuse of marine by-products, linking waste valorization with the development of health-promoting bioactive products. Future optimization of extraction and purification strategies could further enhance yield and bioactivity, promoting the use of these extracts as functional ingredients in innovative therapeutic, nutraceutical, and cosmetic applications. Altogether, these results emphasize the opportunity to transform biological waste into high-value resources within a circular economy framework.

## Figures and Tables

**Table 1:** Concentration of astaxanthin in *C. sapidus* extracts determined by HPLC-DAD, expressed as mg/100 g of dry weight (DW).

*C. sapidus* Extract	AST (mg/100 g DW) ^a^
Ethanol extract	1.269 ± 0.006
Isopropanol extract	1.219 ± 0.015

^a^ Data are expressed as mean (*n* = 3). ± standard deviation (S.D.).

**Table 2:** Total Antioxidant Activity (TAA) of *C. sapidus* extracts and AST.

	TAA	
	DPPH	FRAP
	mgTE/g ± S.D.	mgTE/g ± S.D.
Ethanol extract	0.10 ± 0.01	0.27± 0.02
Isopropanol extract	0.08 ± 0.01	0.11 ± 0.03
AST	0.40 ± 0.09	2.73 ± 0.27

Data are reported as mean ± S.D.= standard deviation (*n* = 3). AST contents in EtOH and IPrOH extracts were 1.02 and 0.98 μg/mL (HPLC, respectively); the AST standard was selected at 1 μg/mL to match the extract levels.

**Table 3:** Antibacterial activity of the AST extract against *S. aureus*.

AST Concentration (µg/Disk)	Inhibition Zone (mm)
≤25	NA
50	12 ± 0.4 ^a^
100	12.7 ± 0.7 ^a^
200	14 ± 1.0 ^b^

NA: No antibacterial activity detected. Data indicate the inhibition zone and standard deviation. Different letters indicate significant differences according to Tukey’s test (*p* < 0.05).

## Data Availability

The original contributions presented in this study are included in the article. Further inquiries can be directed to the corresponding author.
